# Development and Psychometric Evaluation of the Thriving in Nursing Questionnaire (THINQ)

**DOI:** 10.1111/jan.16904

**Published:** 2025-03-17

**Authors:** Judith E. Arnetz, Eamonn Arble, Jackeline Iseler, Michelle Pena, Nicolina Evola, John Vanschagen, Bengt B. Arnetz

**Affiliations:** ^1^ Department of Family Medicine College of Human Medicine, Michigan State University Grand Rapids Michigan USA; ^2^ Department of Psychology Eastern Michigan University Ypsilanti Michigan USA; ^3^ Trinity Health Grand Rapids Michigan USA; ^4^ College of Nursing, Michigan State University East Lansing Michigan USA

**Keywords:** confirmatory factor analysis, exploratory factor analysis, nursing, scale development, thriving

## Abstract

**Aim:**

To develop and evaluate a questionnaire for measuring factors that contribute to thriving at work among nurses.

**Design:**

A cross‐sectional study.

**Methods:**

An online questionnaire was administered in March 2024 to nurses in a community teaching hospital in Michigan, US. Questionnaire content was based on a literature search and was pilot tested among nursing professionals within the hospital system. Questionnaire factor structure was examined with exploratory and confirmatory factor analyses with split‐half sample validation.

**Results:**

Based on exploratory and confirmatory factor analysis, a three‐factor solution presented the best model, with factors comprised of 15 items measuring individual resources (3 items), work resources (6 items) and interpersonal aspects of the nursing work environment (6 items). Reliability estimates for all three factors exceeded 0.80, indicating good internal homogeneity. The questionnaire also demonstrated acceptable split‐half validity and reliability.

**Conclusion:**

The questionnaire presented here provides a potentially useful tool for measuring and evaluating thriving at work among nurses.

**Implications for the Profession and/or Patient Care:**

A better understanding of factors that enhance nurse thriving would lay the foundation for targeted interventions aimed at improving the nursing work environment and nurse well‐being. Enhancing nurse thriving could have a potentially positive impact on patient care.

**Impact:**

This study addressed the need to understand factors that contribute to thriving in nursing work. The questionnaire that was developed revealed a three‐factor solution measuring individual nurse resources, work environment resources and work interpersonal resources. By measuring thriving among nurses, hospitals and other healthcare organisations are taking an important first step in identifying interventions to enhance the nursing work environment, nurse well‐being and potentially the quality of patient care.

**Reporting Method:**

We followed the STROBE checklist in reporting this study.

No patient or public contribution.

**Summary**

- What is already known?
  ○ Nursing work is characterised by high levels of stress associated with burnout and turnover.
  ○ Nurse stress and burnout may jeopardise nurse health and well‐being as well as patient care.
  ○ A growing body of research is focusing on work environment factors that relate to thriving at work, but no existing instruments measure thriving in nursing.
- What this paper adds
  ○ The Thriving in Nursing Questionnaire (THINQ) was developed to measure thriving in nursing work specifically.
  ○ Exploratory and confirmatory factor analyses identified three factors comprised of 15 items measuring individual resources, work resources and work interpersonal resources that contribute to thriving in nursing work.
  ○ The questionnaire demonstrated acceptable split‐half validity and reliability.
- Implications for practice/policy
  ○ Use of the questionnaire has the potential to provide a better understanding of the factors that enhance nurse thriving.
  ○ The questionnaire can help to raise awareness of the key dimensions of thriving and could guide organisational strategies and targeted interventions aimed at improving the nursing work environment and nurse well‐being.
  ○ Efforts that prioritise nurse well‐being and sustainability over burnout can contribute meaningfully to the advancement of the nursing profession.

## 1 Introduction

Nursing work has increasingly been characterised by high levels of stress, burnout and turnover, both in the United States (Shah et al. 2021) and globally (Rink et al. 2023), with conditions exacerbated by the COVID‐19 pandemic (Arnetz et al. 2020; Sexton et al. 2022). A 2019 report from the US National Academies of Sciences, Engineering and Medicine (NASEM) highlighted the risks of burnout for clinician well‐being, patient safety and the quality of patient care (NASEM 2019), leading to the National Academy of Medicine's National Plan for Health Workforce Well‐being (NAM 2022). The plan's vision, ‘…is that patients are cared for by a health workforce that is thriving in an environment that fosters their well‐being as they improve population health, enhance the care experience, reduce costs, and advance health equity, therefore achieving the quintuple aim’ (p. 4). This represents a significant shift from a focus on the negative effects of healthcare work environments on worker health and well‐being to investigation into factors that relate to thriving at work (Moloney et al. 2020).

### 1.1 Thriving at Work

The concept of thriving at work has been studied for decades. Spreitzer et al. (2005) defined thriving as a psychological state characterised by two domains, vitality and learning (Spreitzer et al. 2005). In essence, workers thrive when they experience feeling energised and are continuously learning and developing their knowledge and skills at work (Kleine et al. 2019). Spreitzer et al. (2005) posit that both vitality and learning develop through interactions with others, or social systems, and based on that, they developed a ‘Socially Embedded Model of Thriving at Work’. Importantly, their model distinguishes thriving from resilience, flourishing and subjective well‐being, contending that those constructs are related, but not equivalent to thriving. According to their model, organisations play an important role in each employee's growth, development and health by ‘cultivating thriving’ (Spreitzer et al. 2005, 545). In their meta‐analysis of thriving at work, Kleine et al. (2019) identified antecedents of thriving as individual characteristics (i.e., psychological capital, proactive personality, positive affect, work engagement) and relational characteristics (i.e., supportive coworkers, supportive leadership, perceived organisational support). Outcomes of thriving at work were improved subjective health (e.g., lower burnout), attitude towards work (e.g., work commitment) and work quality (e.g., task performance).

## 2 Background

### 2.1 Thriving in Nursing

Relatively little is known about thriving in healthcare work generally, or in nursing specifically, where the focus of much of the recent literature has been on the growing proportion of burnout in the healthcare workforce (Giusti et al. 2024; Shah et al. 2021; Rotenstein et al. 2023). However, a growing body of research has recently begun to investigate factors related to nurses' thriving at work (Moloney et al. 2020; Mortier et al. 2016; Shen et al. 2024; Yun et al. 2022; Zhai et al. 2023; Zhao et al. 2018; Zhu et al. 2021). Five of the seven referenced studies were cross‐sectional studies of Chinese nurses. In a study of Belgian nurses, Mortier et al. (2016) reported that nurse managers' authentic leadership correlated positively with nurse thriving. Zhu et al. (2021) also found that authentic leadership was a positive predictor of thriving, along with workplace mindfulness, organisational justice and years of experience, while workplace violence was inversely related. Another Chinese study (Zhao et al. 2018) also reported that workplace violence had a negative effect on nurse thriving. Zhai et al. (2023) found that work engagement and affective commitment mediated the relationship between nursing work culture and thriving. Using a time‐lagged survey, Yun et al. (2022) found that high‐performance work systems were positively related to nurse resilience and enhanced thriving. Shen et al. (2024) also studied resilience, reporting that thriving at work partially mediated the relationship between nurse resilience and work performance.

### 2.2 Measuring Thriving at Work

All five Chinese studies and Mortier et al. (2016) utilised the Thriving at Work questionnaire (Porath et al. 2012) that was based on Spreitzer et al.'s (2005) Social Embeddedness Model. Moloney et al. (2020) conducted an integrative literature review on thriving in healthcare organisations and via thematic analysis found that the Social Embeddedness Model can provide support for healthcare managers in developing workplace conditions where nurses can thrive. In a qualitative interview study of 11 Canadian nurses, Jackson (2022) also found that participants' experiences of thriving—vitality, learning, and internal and external factors—were supportive of Spreitzer et al.'s model. The Thriving at Work questionnaire (Porath et al. 2012) is a 10‐item scale with two subscales, Vitality and Learning, each with 5 items that are scored on a 7‐point response scale from 1 (strongly disagree) to 7 (strongly agree). While the questionnaire has been well validated and widely used, there is a lack of consensus on its utility. Abid and Ahmed (2016) contend that thriving encompasses not only vitality and learning but is also defined by cognitive, affective and behavioural dimensions. Both Brown et al. (2017) and Peters et al. (2021) agreed that the two‐dimensionality suggested by Spreitzer et al. (2005) was too narrow, contending that there is a lack of agreement on what thriving is, especially thriving at work. In an effort to develop a Thriving from Work questionnaire, Peters et al. (2021) developed 87 candidate questionnaire items. A questionnaire of that length would likely be difficult to administer among working nurses with busy schedules, who often state that completing surveys simply takes too much time (Timmins et al. 2023). While previous research supports the validity and reliability of the Thriving at Work questionnaire (Porath et al. 2012), the instrument is not specific to the nursing work environment, and using such an instrument may leave many unanswered questions as to factors that relate specifically to thriving in nursing work. This is supported by Brown et al. (2017), who contend that not all instruments measuring thriving at work may be applicable to all contexts and professional groups. In addition, a search of the literature did not find any instruments designed for measuring thriving in healthcare workers generally or nurses in particular.

## 3 The Study

With this background, the aim of the current study was to develop and evaluate a questionnaire for measuring factors that contribute to thriving at work among nurses. A better understanding of factors that enhance nurse thriving would lay the foundation for targeted interventions in hospitals and other healthcare settings.

## 4 Methods

### 4.1 Study Setting

This study was carried out in 2024 at a community teaching hospital in west Michigan, U.S. that is part of a large national hospital system. The hospital has 4953 employees, of which 1553 are registered nurses.

### 4.2 Questionnaire Development

Questionnaire development began with a closer examination of the 10 items in Porath et al.'s (2012) Thriving at Work questionnaire. The five vitality subscale items measured feeling alive and vital, energetic and awake and looking forward to each day at work, while the five learning subscale items concerned learning often and regularly at work. While important and meaningful, these items did not directly capture malleable work factors that could be the foundation for targeted interventions to enhance nurse thriving.

### 4.3 Content

Inspiration for content areas potentially related to thriving came partially from the U.S. Surgeon General's report on Workplace Mental Health and Well‐being (2022). The report describes a framework based on five ‘essential’ components: protection from harm, connection and community, work–life harmony, mattering at work and opportunity for growth (p. 3). These components encompass aspects that our team deemed important and relevant to nursing work; for example, workplace physical and psychological safety, collaboration and teamwork, flexible schedules and work autonomy, sufficient salary, involvement in decision‐making, training and career development and relevant feedback (p.10).

Additional inspiration came from the National Academy of Medicine's (NAM) National Plan for Health Workforce Well‐Being (NAM 2022). The plan describes seven priority areas, one of which is creating and sustaining positive work and learning environments and culture (p. xi). It defines professional well‐being as a ‘function of being satisfied with one's job, finding meaning in work, feeling engaged at work, having a high‐quality working life, and finding professional fulfillment in work’ (p. xiv). These criteria—especially joy, fulfilment and meaning in healthcare work—are considered the bedrock of a thriving healthcare workforce and are prerequisites for safe, high‐quality patient care (NAM 2022, p.1).

**FIGURE 1:**
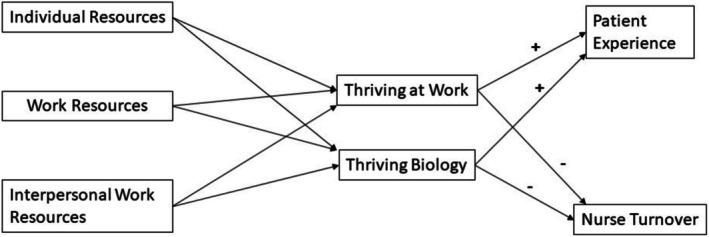
Conceptual model of thriving in nursing work.

With these framework documents as a foundation, we developed a conceptual model of thriving at work that included factors related to the individual nurse, work unit and work interpersonal processes (Figure 1). We further theorised that thriving at work would correlate positively with quality of care and inversely with nurse turnover.

### 4.4 Format

Equally important was that the questionnaire be as short as possible, with items that would be easy for nurses to understand and complete. For this reason, we chose to utilise visual analogue scales (VAS) in place of multi‐item scales. We utilised several items from the Arnetz and Hasson Stress Questionnaire (Hasson and Arnetz 2005; Andersson et al. 2009), an instrument comprised of seven VAS items that was based on the Quality, Work, Competence questionnaire measuring the psychosocial work environment (Arnetz and Arnetz 1996; Arnetz and Hasson 2007). Research has found that these VAS items can be used in place of Likert‐type items (Hasson and Arnetz 2005) and have sound psychometric properties (Andersson et al. 2009).

### 4.5 Pilot Questionnaire

**FIGURE 2:**
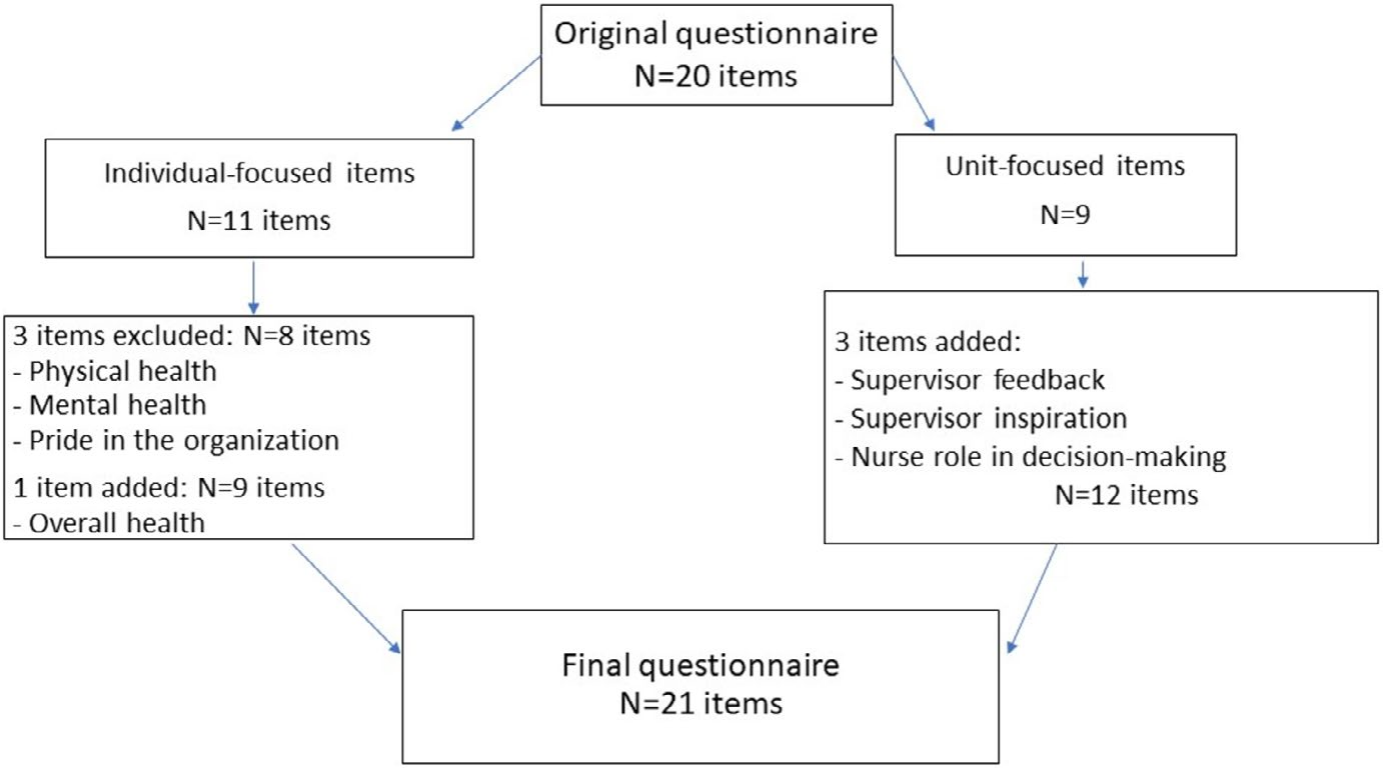
Flow chart describing development of the questionnaire from pilot version to final version.

The first version of the questionnaire was comprised of 20 VAS items measured on a scale from 0 to 10, with anchor terms very poor—very good, completely disagree—completely agree or never—always. The 20‐item questionnaire encompassed 11 items related to the individual and 9 unit‐focused items. The draft questionnaire was shared with our hospital stakeholder partners and deemed acceptable in terms of the questions being relevant and easily understandable. Our hospital partners shared the survey with their Nursing Practice and Environment Council who also reviewed the survey. Based on this iterative process, three items related to physical health, mental health and pride in the organisation were removed, and three items related to supervisor feedback, supervisor encouragement and nurse role in decision‐making were added. A single question, ‘How is your overall health right now?’ replaced the two separate items asking about physical health and mental health, respectively. For several items, the formulation was slightly modified after council review. One such item with an individual focus, ‘I provide high‐quality care for my patients’, became unit‐focused, ‘Our unit has the necessary staffing to provide high‐quality care for our patients’. A flow chart summarising the development of the questionnaire from the pilot version to the final version is presented in Figure 2. The result was a survey comprised of 21 single‐item VAS questions. The survey also included 7 demographic questions measuring hospital unit, shift, years of nursing experience, age, gender, Hispanic/Latino origin and race/ethnicity.

### 4.6 Study Participants

All registered direct care nurses employed by the hospital (*n* = 1100) were eligible to participate in the ‘Nurse Thriving and Well‐Being Study’. An invitation to participate was sent via email to hospital nurses by nurse leadership. The email explained the purpose of the survey was to investigate factors that contribute to nurse thriving and well‐being. Nurses would respond anonymously and were assured that individual responses could not be linked to their names or emails. Those nurses agreeing to participate could access the electronic Qualtrics survey by either scanning the QR code or using the weblink provided. The first page of the survey explained that participation was voluntary, and that completing the survey constituted nurses' consent to participate. Recruitment was also conducted via posters advertising the survey that were mounted in areas of the hospital frequented by nurses.

### 4.7 Data Collection

The survey was open over a 5‐week period from late March to early May 2024. Weekly reminders about the survey were posted by nursing leadership on the hospital's nursing FaceBook page over the 5‐week study period. Of the 1100 surveys distributed, a total of 252 were returned, representing a 23% response rate. Among questionnaire respondents, 92.1% identified as female, 93.9% as Caucasian, with 32.7% aged 25–34 and 24.8% aged 35–44. The majority (76.1%) worked day shift, and 50% had over 10 years' experience as a nurse. Based on the gender (92.1% female) and ethnic identification (93.9% Caucasian) of respondents, the study population differed slightly from the total population of hospital nurses: 87.7% female, *χ* ^2^ = 4.95, *p* < 0.05; 86.2% Caucasian, *χ* ^2^ = 21.5, *p* < 0.001. Demographic characteristics of the study sample are summarised in Table 1. Of note, approximately 15% of demographic variables were systematically missing. A total of 38 respondents started but did not complete the questionnaire; of those, 4 stopped responding after question 4, and the remaining 28 answered all questions except the demographic items, which came at the end.

**TABLE 1:** Demographics of the study sample (*N* = 252).

Demographic variable	Frequency (*n*)	Percent (%)
*Age (years)*		
18–24	21	9.8
25–34	70	32.7
35–44	53	24.8
45–54	39	18.2
55–64	21	9.8
65–74	6	2.8
Prefer not to answer	4	1.9
Total	214	100
Missing	38	15.1
*Gender*		
Male	15	7.0
Female	197	92.1
Prefer not to answer	2	0.9
Total	214	100
Missing	38	15.2
*Race/ethnicity*		
White/Caucasian^a^	201	93.9
Asian	3	1.4
Black/African American^a^	1	0.5
American Indian/Alaska Native	1	0.5
Prefer not to answer	8	3.7
Total	214	100
Missing	38	15.1
*Usual shift*		
Day	162	76.1
Mid‐day	11	5.2
Night	40	18.8
Total	214	
Missing	39	15.5
*Years as a Nurse (years)*		
< 5	44	20.8
5–10	62	29.2
> 10	106	50.0
Total	212	100
Missing	40	15.9

*Note:* Demographic description of the original sample (*N* = 252) is summarised from self‐report responses to categorical scales.

^a^ Respondents within this group self‐identified as Hispanic, Latino or of Spanish origin: *n* = 3 Hispanic White Caucasian, and *n* = 1 Hispanic Black/African American.

### 4.8 Ethical Considerations

The study was approved by the Institutional Review Boards at Michigan State University (Study ID 00009623) and Trinity Health Grand Rapids Hospital (Study ID 24‐0122‐4).

### 4.9 Statistical Analysis

All factor analyses were completed in MPlus (v8.8); descriptive statistics and scale reliability were estimated with SPSS (v28). As a first step, a correlation matrix was created to study the inter‐item correlations of 18 of the questionnaire items. Analyses excluded three items measuring feeling fulfilled at work, feeling that work is meaningful and feeling joyful at work, as these are part of the National Academy of Medicine's (NAM) definition of thriving at work (NAM 2022). The 7 background/demographic variables were also excluded. Bartlett's test of sphericity and the Kaiser–Meyer–Olkin measure of sampling adequacy (KMO) were used to assess the factorability of the correlation matrix. Exploratory factor analysis with principal axis factoring and geomin rotation (30 random starts) was used to examine the structure of relationships between the items and for factor extraction. Subscale (factor) independence was examined by correlation analysis. Internal reliability of the scales was measured using McDonald's omega (*ω*). Criteria for item inclusion and factor creation were (1) item loadings of 0.30 or higher (Rattray and Jones 2007), (2) a minimum of 3 items and (3) a reliability coefficient of 0.70 or greater. Additional analysis was completed by confirmatory factor analysis with principal axis factoring. The exploratory and confirmatory factor analyses were completed with a sample *N* = 245. Of the 252 responding nurses, *n* = 194 (77%) had complete responses to all items included in the factor analysis; *n* = 58 (23%) were missing at least 1 response. Missing data frequency ranged from 2.8% up to 17.5% per item, and *n* = 245 had at least 1 response on the items for analysis; data were supported to be missing at random (Little's *χ* ^2^ (233) = 243.11, *p* = 0.31). Therefore, responses for *n* = 245 were included, and the factor analyses were fit with full information maximum likelihood estimation, which provides unbiased estimates under these missing data conditions (Little et al. 2014; Mazza et al. 2015). Optimising the analysis to retain as many cases as possible, opposed to listwise deletion for complete data, improves the external validity of the parameter estimates under these conditions (Little et al. 2014).

For factor identification as a stage of scale development, the sample was split in half with random assignment to two groups. Sample 1 (*n* = 122) was used for exploratory factor analysis with oblique rotation (i.e., allowing factors to correlate and cross‐loadings of items across all factors). Following the exploratory procedure, the best factor model solution was re‐specified in confirmatory factor analysis without cross‐loading and including correlated factors. In instances of items with significant cross‐loadings, the factor with the highest loading was retained for additional testing in the confirmatory factor analysis procedure. The confirmatory factor analysis estimates were compared between Sample 1 and Sample 2 (*n* = 123) with a grouped modelling procedure to test replication of the factor loadings. Factor loadings were compared between samples with bias‐corrected bootstrapped 95% confidence intervals (10,000 draws), which, if overlapping between samples, supports comparable loadings. In confirmatory factor analysis, the first item on each factor was specified with a loading equal to 1 to identify the factor. Model fit was assessed by multiple indices. In the exploratory analysis, decreasing eigenvalue indicates improvement in the factor model to explain more variance in the indicator items. In all model testing, model *χ* ^2^ non‐significance indicated fit of the structural model; however, this index is biased in large samples and models with large degrees of freedom, and so *χ* ^2^/df is prioritised, and values below 5 support good model specification. Significant change in chi‐squared indicates the improvement in fit with increasing factor structure. CFI (> 0.9), RMSEA (< 0.1) and SRMR (< 0.1) are additional fit indices for the structural model. Confirmatory factor solutions were examined for internal consistency with McDonald's omega (*ω*), for which values > 0.8 were considered good. As a final consideration, equivalent factor loadings and correlations between groups of nurses of different professional experience levels were examined with a grouped modelling procedure (*n* = 212 had responses to the experience item for analysis), including group comparisons by approximate *z*‐test and an adjusted *α* = 0.01 to mitigate potential familywise error in the exploratory analysis.

## 5 Results

### 5.1 Step 1: Exploratory Factor Analysis

All eligible items were submitted for exploratory factor analysis to fit models with 1 to 4 factors in Sample 1. The model solution was chosen based on decreasing eigenvalue and improvement in model fit, while prioritising parsimony. Reviewing the model fit summary in Table 2, there is increasing model improvement with additional factors added. A 3‐factor solution is supported by both improvement in model fit and the reduction in eigenvalue that is negligibly improved with 4 factors. It should be noted that although the 4‐factor solution has the best structural model fit, when weighed against the incremental additional variance explained indicated by the eigenvalues and prioritising parsimony, the 3‐factor solution is favourable. The factor weights for the exploratory factor analysis with the 3‐factor solution in Sample 1 are summarised in Table 3.

**TABLE 2:** Summary of model fit by exploratory factor analysis.

Fit index	1 Factor	2 Factor	3 Factor	4 Factor
Eigenvalue	7.582	1.845	1.409	1.152
*χ* ^2^, *p*‐value	507.17, *p* < 0.001	335.08, *p* < 0.001	240.73, < 0.001	178.69, < 0.001
Δ *χ* ^2^	—	172.09, *p* < 0.001	94.35, *p* < 0.001	62.03, *p* < 0.001
*χ* ^2^/df	3.76	2.84	2.36	2.05
CFI	0.67	0.81	0.88	0.92
RMSEA	0.15	0.12	0.11	0.09
SRMR	0.1	0.08	0.06	0.05

**TABLE 3:** Exploratory factor analysis item loadings.

Item	Factor 1	Factor 2	Factor 3
Q1_Energy	**0.738** *	−0.024	0.233
Q2_Concentrate	**0.864** *	0.038	−0.030
Q3_Health	**0.751** *	0.023	0.052
Q5_PosWorkClimate	0.080	0.376*	**0.440** *
Q6_ProfCompetence	0.257*	**0.354** *	0.157
Q7_Skills	−0.020	**0.764** *	−0.170
Q8_Staffing	0.053	**0.601** *	−0.103
Q9_Efficiency	0.021	**0.580** *	0.217
Q10_ToughSituation	0.193	**0.647** *	−0.001
Q11_Team	−0.106	**0.565** *	0.288
Q12_Feedback	−0.051	0.026	**0.957** *
Q13_Inspire	0.006	−0.009	**0.948** *
Q14_DecisionMaking	0.109	0.155	**0.547** *
Q16_Spirituality	0.222	−0.083	**0.288** *
Q17_Salary	0.250*	0.168	**0.288** *
Q18_ProbsIssues	−0.008	0.357*	**0.542** *
Q20_Memory	**−0.457** *	−0.082	0.209
Q21_Stress	−0.270*	**−0.350** *	−0.028

*Note:* Loadings estimated with oblique rotation are reported in Sample 1. Bolded values indicate the highest loading that is retained for further analysis in the confirmatory procedure.

* Indicates significance *p* < 0.05.

### 5.2 Step 2: Confirmatory Factor Analysis With Split‐Half Sample Validation

The 3‐factor solution, excluding cross‐loadings for items, was respecified as a confirmatory factor analysis in Sample 1 and Sample 2 using a grouped modelling procedure, with the purpose of replication of the factor loadings. All loadings were similar between the samples based on a comparison of bootstrapped 95% confidence intervals that overlapped (Table 4). The item Q16_Spirituality had consistently small loadings, and the Factor 3 specification accounted for ≤ 10% of variance in the response to the item. Therefore, this item was removed from further analysis. Factor 1 had poor reliability due to the inclusion of Q20_Memory (McDonald's *ω*: Sample 1 = 0.53; Sample 2 = 0.55), which also had small variance explained by the factor structure (*R* ^2^ ≤ 0.10). Factor 2 also had poor reliability due to the inclusion of Q21_Stress (McDonald's *ω*: Sample 1 = 0.69; Sample 2 = 0.79). Therefore, these items were removed. The resulting factors had excellent reliability: Factor 1 (Sample 1 = 0.87, Sample 2 = 0.87), Factor 2 (Sample 1 = 0.81, Sample 2 = 0.89), and Factor 3 (Sample 1 = 0.87, Sample 2 = 0.85).

**TABLE 4:** Standardised factor loadings with split‐half sample validation.

Item	Factor 1				Factor 2				Factor 3			
	Sample 1		Sample 2		Sample 1		Sample 2		Sample 1		Sample 2	
	Loading	*R* ^2^	Loading	*R* ^2^	Loading	*R* ^2^	Loading	*R* ^2^	Loading	*R* ^2^	Loading	*R* ^2^
Q1_Energy	0.857 (0.77, 0.92)	0.734	0.863 (0.78, 0.93)	0.745								
Q2_Concentrate	0.857 (0.74, 0.94)	0.734	0.826 (0.74, 0.89)	0.682								
Q3_Health	0.757 (0.65, 0.84)	0.573	0.772 (0.69, 0.85)	0.597								
Q20_Memory	−0.298 (−0.47, −0.12)	0.09	−0.330 (−0.48, −0.15)	0.109								
Q6_ProfCompetence					0.572 (0.41, 0.73)	0.327	0.692 (0.55, 0.81)	0.479				
Q7_Skills					0.491 (0.32, 0.69)	0.241	0.643 (0.48, 0.76)	0.413				
Q8_Staffing					0.635 (0.53, 0.74)	0.404	0.696 (0.48, 0.83)	0.485				
Q9_Efficiency					0.764 (0.67, 0.86)	0.583	0.825 (0.63, 0.92)	0.68				
Q _ToughSituation					0.639 (0.46, 0.80)	0.408	0.734 (0.61, 0.84)	0.539				
Q11_Team					0.674 (0.49, 0.82)	0.454	0.734 (0.61, 0.83)	0.539				
Q21_Stress					−0.500 (−0.65, −0.36)	0.25	−0.485 (−0.61, −0.35)	0.235				
Q5_PosWorkClimate									0.714 (0.57, 0.81)	0.51	0.69 (0.54, 0.80)	0.476
Q12_Feedback									0.932 (0.80, 0.98)	0.868	0.893 (0.72, 0.96)	0.797
Q13_Inspire									0.936 (0.84, 0.97)	0.877	0.89 (0.71, 0.96)	0.793
Q14_DecisionMaking									0.705 (0.57, 0.81)	0.497	0.663 (0.54, 0.77)	0.439
Q16_Spirituality									0.32 (0.15, 0.49)	0.102	0.3 (0.15, 0.44)	0.09
Q17_Salary									0.523 (0.35, 0.656)	0.273	0.475 (0.33, 0.60)	0.225
Q18_ProbsIssues									0.736 (0.58, 0.85)	0.541	0.709 (0.56, 0.82)	0.503

*Note:* Standardised loading with bootstrapped 95% confidence intervals (LL, UL) is reported from a grouped modelling procedure to compare Sample 1 and Sample 2. All loadings are significant, *α* = 0.05.

### 5.3 Step 3: Final Confirmatory Factor Analysis

Based on the summarised evidence, items Q16_Spirituality, Q20_Memory and Q21_Stress were removed from the final model. This resulted in a 3‐factor solution that had acceptable model fit in the total sample: *χ* ^2^ = 424.82, *p* < 0.001; *χ* ^2^/df = 4.88; CFI = 0.83, RMSEA = 0.13, SRMR = 0.09. Based on item content, Factor 1 was named Individual Resources, Factor 2 Work Resources and Factor 3 Work Interpersonal Resources. All factors had good internal consistency measured by McDonald's omega (*ω*): Factor 1 *ω* = 0.87, Factor 2 *ω* = 0.83 and Factor 3 *ω* = 0.88, and all items had significant loadings (Table 5). Factors were intercorrelated: Factor 1 with Factor 2 (*r* = 0.66, *p* < 0.001) and Factor 3 (*r* = 0.53, *p* < 0.001), and Factor 2 with Factor 3 (*r* = 0.71, *p* < 0.001). Because a 3‐factor solution fit better than a 1‐factor solution and item cross‐loadings across factors were constrained, correlations among the factors most likely reflect associations among the latent constructs opposed to redundant measurement among items. An overview of the 3 final factors with their component items in the Thriving in Nursing Questionnaire (THINQ) is presented in Table 6.

**TABLE 5:** Final factor model description of standardised loadings in the total sample (*n* = 245).

Item	Factor 1	Factor 2	Factor 3	*R* ^2^
	Individual resources	Work resources	Work interpersonal resources	
Q1_Energy	0.87			0.76
Q2_Concentrate	0.83			0.69
Q3_Health	0.76			0.58
Q6_ProfCompetence	0.65			0.42
Q7_Skills		0.60		0.36
Q8_Staffing		0.64		0.41
Q9_Efficiency		0.78		0.61
Q10_ToughSituation		0.71		0.50
Q11_Team		0.71		0.51
Q5_PosWorkClimate			0.69	0.48
Q12_Feedback			0.92	0.85
Q13_Inspire			0.93	0.86
Q14_DecisionMaking			0.68	0.47
Q17_Salary			0.49	0.24
Q18_ProbsIssues			0.71	0.51

**TABLE 6:** Item content for the three factors in the Thriving in Nursing Questionnaire (THINQ).

Individual resources (3 items)	Work resources (6 items)	Work interpersonal resources (6 items)
Q1 How is your energy right now?	Q6 In my job, I develop my professional competence	Q5 There is a positive work climate in my unit
Q2 How is your ability to concentrate right now?	Q7 I have the necessary skills to provide high‐quality patient care	Q12 My supervisor provides me with constructive feedback
Q3 How is your overall health right now?	Q8 Our unit has the necessary staffing to provide high‐quality care for our patients	Q13 My supervisor inspires me to do the best job I can
	Q9 Our unit runs efficiently	Q14 I play a role in the decision‐making process on my unit
	Q10 I am able to handle a tough situation at work	Q17 I am satisfied with my salary
	Q11 Our team works well together	Q18 In my unit, people are able to bring up problems and tough issues

### 5.4 Step 4: Comparison of Factor Structure by Experience Level of the Nurse

As a final consideration, the final factor structure was tested for equivalent factor loadings across groups of nurses with different experience levels: level 1 (less than 5 years, *n* = 44), level 2 (5–10 years, *n* = 62), level 3 (over 10 years, *n* = 106). This analysis was on a total sample size of *N* = 212, due to missing data on reported experience level. The model had modest fit: *χ* ^2^ (295) = 732.60, *p* < 0.001 (Exp 1 = 216.05, Exp 2 = 226.33, Exp 3 = 290.21); CFI = 0.78; RMSEA = 0.15, SRMR = 0.16. Overall, the factor loadings were similar across groups (Table 7) and fell within the range of the original randomised split‐half samples, and there was significant variability in factor scores within each group. Together, this supports the use of this scale with nurses of all experience levels. A few notable variations in the factor structure were observed that bring to light interesting questions of self‐reported perceptions of these constructs with more professional experience. Perceptions of adequate staffing carried greater weight at lower experience levels, while ratings of efficiency were highest weighted at mid‐career experience. Correlations among factors were also increased in effect size with greater experience.

**TABLE 7:** Description of factor loadings across groups of nurses of increasing professional experience (*n* = 212).

Item	Exp = 1 (< 5 years)			Exp = 2 (5–10 years)			Exp = 3 (> 10 years)		
	*N* = 44			*N* = 62			*N* = 106		
	Factor 1	Factor 2	Factor 3	Factor 1	Factor 2	Factor 3	Factor 1	Factor 2	Factor 3
Q1_Energy	1			1			1		
Q2_Concentrate	**0.86**			**0.86**			**0.86**		
Q3_Health	**0.60**			**0.60**			**0.60**		
Q6_ProfCompetence		1			1			1	
Q7_Skills		**1.03** ^†^			0.26^†^			**0.69**	
Q8_Staffing		**2.31** ^•^			**1.99** ^◊^			**1.09** ^◊^	
Q9_Efficiency		**1.59**			**2.15** ^◊^			**1.32** ^◊^	
Q10_ToughSituation		**1.00**			**0.77**			**1.07**	
Q11_Team		**0.87**			**1.38**			**1.15**	
Q5_PosWorkClimate			1			1			1
Q12_Feedback			**1.97**			**1.71**			**2.21**
Q13_Inspire			**1.83**			**1.95**			**2.32**
Q14_DecisionMaking			**1.37**			**1.09**			**1.96**
Q17_Salary			**0.74**			**0.85**			**1.63**
Q18_ProbsIssues			**0.65** ^•^			**1.14** ^•^			**1.74**
*Correlations*									
Factor 1–2		**1.15**			**0.69**			**1.17**	
Factor 1–3	0.62			**1.00**			**0.97**		
Factor 2–3	0.23			**0.82**			**0.81**		
Mean	0	0	0	−0.10	−0.34	0.22	0.51	0.03	**0.42**
Variance	**2.53**	**1.15**	**1.11**	**2.34**	**1.17**	**1.68**	**3.51**	**1.16**	**0.97**

*Note:* Unstandardised loadings are reported. Values bolded indicate significance within group (*p* < 0.05). The loading of the first item for each factor was specified to be fixed at 1 for all groups, and therefore, no significance testing is available for the unstandardised loadings. Between‐group comparisons in estimated factor loadings were made by approximate *z*‐test (*p* ≤ 0.01): ^†^significant difference of Exp 1 vs. Exp 2; ^•^significant difference of Exp 1 vs. Exp 3; ^◊^significant difference of Exp 2 vs. Exp 3. Group means were estimated relative to Exp 1 (centred mean = 0).

## 6 Discussion

The aim of this study was to develop and psychometrically evaluate a questionnaire for measuring thriving in nursing work. Based on exploratory and confirmatory factor analysis, a three‐factor solution presented the best factor model, with factors comprised of 15 items measuring individual resources (3 items), work resources (6 items) and interpersonal aspects of the nursing work environment (6 items). Reliability estimates for all three factors exceeded 0.80, with two exceeding 0.85, indicating good internal homogeneity. The questionnaire also demonstrated acceptable split‐half reliability. Content validity was established through literature searches and a review of the pilot questionnaire by nurse professionals within the hospital system. Inter‐scale correlations between the three factors, a measure of construct validity, ranged from 0.53 to 0.71, indicating moderate subscale independence but suggesting some potential overlap. However, based on the item content of each subscale and the confirmatory factor analysis, the three‐factor solution was deemed the best model. Results also supported split‐half validity, another example of construct validity and the questionnaire's ability to distinguish between groups of nurses based on years of experience, an indicator of discriminant validity.

The three domains resulting from this analysis confirmed our conceptual model suggesting that thriving in nursing work encompasses both individual and work‐related factors. This is in line with Brown et al. (2017) whose definition of thriving encompassed both personal and contextual ‘enablers’ (p. 170). Our identification of both individual and interpersonal characteristics also supports Kleine et al. (2019), whose meta‐analysis found individual and relational antecedents of thriving.

### 6.1 Individual Resources Factor

Kleine et al.'s (2019) meta‐analysis defined individual thriving characteristics as, for example, psychological capital, proactive personality, positive affect and work engagement. However, the individual resource items in our questionnaire (energy, ability to concentrate and overall health) are closer in nature to Porath et al.'s (2012) vitality subscale. While health and energy are in line with Porath et al.'s vitality measure, the ability to concentrate is a more specific measure. Recent research has shown that nurses' inability to concentrate, or cognitive failure, at work has been associated with extreme stress (Arnetz et al. 2021), substance use (Arble et al. 2023) and workplace violence (Arnetz et al. 2024). Thus, the ability to concentrate may be a reflection of a well‐functioning individual who is thriving in a nursing environment.

### 6.2 Work Resources Factor

The Work resources subscale includes two items (developing professional competence and having the necessary skills) that are similar to items in Porath et al.'s (2012) learning subscale. Skills and competence development are components in the ‘Opportunities for Growth’ aspect of the U.S. Surgeon General's framework (2022) and have been shown to be important for nurses' well‐being (Arnetz et al. 2019; Hasson and Arnetz 2008). However, the Work resources subscale includes additional items reflective of the nursing work environment, including necessary staffing for high‐quality care, unit efficiency and ability to handle tough situations. Nurse staffing shortages have long been identified as a source of stress among nurses (Jackson et al. 2007; Shah et al. 2021), while work efficiency has been inversely associated with nurses' experiences of workplace bullying and violence (Arnetz et al. 2018; Viotti, Essenmacher et al. 2018; Viotti, Converso et al. 2018). It has been suggested that nurses with the ability to handle tough situations, that is, resilience, are more likely to thrive at work (Jackson et al. 2007).

### 6.3 Work Interpersonal Resources Factor

In Kleine et al.'s (2019) meta‐analysis of thriving at work, relational characteristics, such as supportive coworkers, leaders and perceived organisational support, closely reflect the items in our Work interpersonal resources subscale. Additional items in this subscale include playing a role in decision‐making and psychological safety. In previous research, psychological safety among nurses, that is, the ability to speak up and discuss difficult issues with management and coworkers (Edmondson 1999), was inversely associated with work stress and positively associated with dehydroepiandrosterone sulfate (DHEA‐S), a biomarker that may signal effective stress management (Arnetz et al. 2019; Cho et al. 2019). Psychological safety has been linked to employee thriving in relation to various leadership behaviours and employee creativity (Yang et al. 2021) and taking‐charge behaviour (Zeng et al. 2020). However, those studies were conducted in other industries, and not among nurses. Engaging nurses in workplace decisions is an element of ‘mattering at work’ that is one of the five essential aspects of the U.S. Surgeon General's Workplace Mental Health and Well‐being framework (U.S. Surgeon General 2022). Satisfaction with salary loaded on the work interpersonal resources subscale and is also an element in ‘mattering at work’ in the U.S. Surgeon General's framework (2022). This factor may be indicative of a nurse's ability to discuss the matter of financial compensation with their supervisor, which would explain the inclusion of this item in the interpersonal resources factor. Salary has in previous research been associated with nurses' turnover (Gardulf et al. 2005), job satisfaction (Atefi et al. 2014) and quality of working life (Hsu and Kernohan 2006). Gardulf et al. (2005) found that nurses who quit their jobs had a significantly lower understanding of the factors determining their salary compared to their colleagues who remained on the job. Communication about salary with one's supervisor could be a factor in nurse motivation to remain and thrive on the job.

In summary, our Thriving in Nursing Questionnaire (THINQ) encompasses aspects of vitality in the Individual resources subscale and aspects of learning in the Work resources subscale, and in these aspects is similar to the Thriving at Work questionnaire developed by Porath et al. (2012). However, as theorised, the THINQ also encompasses components more specific to the nursing work environment, including nurse staffing, workplace psychological safety, teamwork, unit efficiency, supervisor feedback, decision‐making and sufficient salary. Porath et al.'s (2012) questionnaire is focused on individual factors but lacks the relational component as well as factors reflective of the work environment aside from learning opportunities.

### 6.4 Questionnaire Feasibility

A key aspect of questionnaire development is the instrument's feasibility, that is, whether it will be easily understood and completed. Less than one quarter of the study population replied to the survey, which might raise questions about feasibility. However, similarly low response rates on surveys of nurses have been reported (L'Ecuyer et al. 2023; Timmins et al. 2023). Research has shown that 40% of a nurse's workday is devoted to demands of the healthcare delivery system that are not directly patient‐related (Krichbaum et al. 2007). It has been suggested that responding to surveys is the type of task included in that 40%, and nurses may choose not to prioritise surveys over focusing on their patient care responsibilities (Kramer et al. 2009). While the research team did send out weekly reminders to hospital nurses, we did not provide incentives in the form of gift cards or other reimbursements, which may have enhanced response.

### 6.5 Limitations

While the psychometric properties of the questionnaire were acceptable, this was the first and only time that the instrument has been used at a single site, and further studies are needed to confirm its validity, reliability and feasibility. In addition to the low response rate, it was not possible to investigate whether respondents differed from non‐respondents in any way that might have introduced bias into the study. Based on the gender and ethnic identification of respondents, there was a small but statistically significant difference between the study sample and the hospital population. However, there is no scientific evidence that there are systematic differences between respondents and non‐respondents on workplace surveys (Thomsen 2000). The study was cross‐sectional, and all data were self‐reported, increasing the risk of bias, such as social desirability, in nurses' responses. Future studies incorporating nurse interviews and/or focus groups on thriving at work could help to confirm the questionnaire's validity.

## 7 Conclusion

The questionnaire presented here provides a potentially useful tool for measuring and evaluating thriving at work among nurses. Use of this questionnaire could provide hospitals with thriving ‘profiles’ of nurses generally, as well as on different units, an important first step in identifying interventions aimed at enhancing the nursing work environment and nurse well‐being. By measuring and fostering awareness of these key dimensions of thriving, the questionnaire has the potential to guide organisational strategies that prioritise nurse well‐being and sustainability over burnout, thereby contributing meaningfully to the advancement of the nursing profession.

## Conflicts of Interest

The authors declare no conflicts of interest.

## Data Availability

The data that support the findings of this study are available from the corresponding author upon reasonable request.
